# 

**Published:** 1860-06

**Authors:** 


*The Scientific American* remarks that the late prize fight would afford the London *Times* a good text for one of its interesting articles on the physical decline of the Americans.

---

A new Medical College has been organized at Leavenworth City, Missouri. It is to have ten professorships.

